# On the Bodily Basis of Human Cognition: A Philosophical Perspective on Embodiment

**DOI:** 10.3389/fnhum.2021.745095

**Published:** 2021-12-08

**Authors:** Amitabha Das Gupta

**Affiliations:** University of Hyderabad, Hyderabad, India

**Keywords:** embodiment, disembodiment, categorisation, metaphor, colour vision

## Abstract

This paper seeks to show that human cognition cannot be characterised purely in mentalistic term. It has a bodily basis and cognition is thus the product of the interplay between mind, body, and brain. This is how the idea of embodiment and its importance is realised and gets its foothold in both philosophy and cognitive science. This brings a radical change introducing a new framework for philosophy and cognitive science. In this new change philosophy and cognitive science have a special role to play which this paper seeks to explore. Philosophy in its capacity as a conceptual inquiry provides justification for human embodiment on a conceptual ground whereas cognitive science provides the same on an empirical and experimental ground. This brings the two disciplines closer resulting into a new field of inquiry which can be best described as the interface between philosophy and cognitive science. An important consequence that follows from this alignment is that the traditional epistemological distinction between the a priori and the empirical can no longer be rigidly maintained.

## Conceptual Mapping of the Paper

It is true, but nevertheless trivially true, that we cannot conceive of human beings to be without bodies. Hence the idea of embodiment is not an abstract idea. On the contrary, it is an existential fact of our lived experience for which no justification is required. However, what is certainly required is to have a definite idea about how mind and body interact with each other. The knowledge of mind–body interaction will reveal certain startling facts about ourselves. It will reveal that the various facets of experience that we have while interacting with the environment and the significance that we derive thereby from it depends on the kind of bodies that we have. That means, the way we look at the world is determined by the way we are embodied. If, on the other hand, we were embodied differently we would not see the world in the same way as we presently do. Considering this overwhelming importance of embodied perspective, it is not difficult to imagine that our cognitive activities too are shaped and guided by our bodily characteristics and activities. There is a structural connexion to be found between our bodily activities and the higher order cognitive operations that we perform. Our understanding of abstract concepts and the reasoning that we do with them have their roots in our bodily experiences. This shows that it is impossible to make a clear-cut distinction between the physical and the mental. Their overlapping nature becomes obvious from the way they function. In view of this, there is a growing realisation that the earlier idea of the autonomy of the mental can no longer be defended. Its characterisation as autonomous and thus disembodied is not so obvious as it was held to be since the time of Cartesian philosophers.

However, the question arises, is this true to say that due to the rise of the embodied conception of the mind, the autonomy of the mental cannot be held any longer? Is the autonomy of the mental so remote? If it is not, then how tenable is the alternative view concerning the embodied conception of the mind? The recent intellectual/philosophical tradition is an evidence to this since it holds a position which is contrary to the embodied conception of mind and cognition. Accordingly, cognition is essentially viewed as mental. This nature is evidently found from cognitive activities that we perform. They are performed in complete isolation from bodily activities because mental activity forms a separate order of activity. This speaks for the disembodied nature of mind which makes the process of cognition devoid of any reference to body. Many of the recent trends in contemporary thought, such as, analytic philosophy, cognitive science, artificial intelligence, etc., carry out their respective research programmes on the line of this assumption subscribing to the disembodied view of cognition which is directly a conceptual outcome of the disembodied view of the mind. There is an inseparable relationship existing between the two that leads to the formulation of the cognitivist paradigm where the act of cognising is explained in terms of the manipulation of formal/computational rules that the mind/brain is endowed with. In this context, to bring the analogy of computer to explain the workings of the mind is a landmark showing a new direction of research in the areas of philosophy of mind and cognitive science. The disembodied approach thus becomes the most plausible approach to understand the various aspects of cognition and cognitive abilities. It is, however, important to make a clarification that the recent disembodied approach to mind and cognition does not adhere to the Cartesian legacy of the mind/body dualism at the background of its theoretical enterprise. Without assuming any such dualistic metaphysics, its concern is to show that cognition and our cognitive abilities that result from our cognitive competence is essentially a mental competence.

This paper takes a critical stance, as indicated earlier, to the idea of cognition viewed as mental and in this respect particularly to the idea of cognitive operations which are defined in terms of the computational abilities of the mind/brain. They constitute the disembodied approach to cognition. As against this disembodied approach, the main line of the argument structure of the paper is to explore the epistemological significance of the embodied approach to cognition with a view to show that body has a constructive role to play in shaping our cognition and thus its role cannot be ignored. Considering this objective, its main thrust will be to show that various cognitive operations that we perform, such as, conceptualising, reasoning, understanding, etc., have their roots in bodily behaviour. In this context, an elaborate argument will be given to show how our sensorimotor experiences will be especially important in acquiring cognition.

In view of the above conceptual layout, the present paper proposes to have three sections. The first section will be concerned with the present scenario existing in philosophy. In this respect, we will be particularly concerned with the disembodied view of meaning and mind as held in contemporary analytic philosophy. The primary preoccupation of analytic philosophy is to engage with the study of meaning or meaning of concepts. To decipher the meaning of concepts involves bringing out the conceptual thought embodied in concepts. But these embedded thoughts can be shown to have their basis in our bodily experience. In analytic philosophy this crucial aspect of the bodily basis of meaning has been completely ignored because by nature meaning has been thought to be disembodied. The disembodied view of meaning later provided the basis for the computational theory of mind which too conceived mind as disembodied in nature and the faculty of reasoning was conceived as having algorithmic-like structure that made the entire process mechanical. Human embodiment thus appears to have no role in our cognitive activities. This is the disembodied perspective—the existing scenario in philosophy, that finds our cognitive activities to be essentially mentalistic. Against this backdrop, we will introduce the idea of human embodiment in the second and third sections.

The idea of human embodiment has been discussed in the second and third sections at two levels. The task of the second section will be to talk about the role of embodiment in philosophy. For this purpose, it will offer philosophical arguments justifying how cognition necessarily involves bodily activities. In this connexion we will specially discuss the role of sensory motor system in acquiring knowledge and skill. Maurice Merleau-Ponty's work on perception in this context will be central to this discussion. His pioneering work on perception offers a new line of arguments and justification for a philosophical framework of embodied cognition. The third section, on the other hand, is more specific in terms of its objective. It will be thus, as mentioned earlier, concerned with certain specific cognitive abilities. We, humans, acquire these abilities due to certain motor abilities of the body that determine how we experience things and how we understand the world. This, in turn, provides the basis for human conceptualisation which are of various kinds. In this context, the three specific instances of conceptualisation will be briefly discussed to address and explain the issue that human conceptualisation is by nature embodied conceptualisation. The issues to be discussed are:

1. How do we understand basic level categories in language?
2. How do we form colour concepts and categories? and
3. How do metaphors that form on the basis of bodily experience shape our conceptual system expressed through language?

### I. The Disembodied Conception of Meaning and Mind

The contemporary Anglo-American philosophy has been largely dominated by the linguistic approach which is also known as the analytic approach to philosophy. As against its metaphysical past, philosophy in the contemporary period is practised on a completely different line of assumption. Its main concern has been linguistic in nature because philosophical problems are now perceived differently. They are viewed as problems arising out of language or meaning. The study of meaning is thus placed at the forefront of philosophical pursuit. Considering this, the logical analysis of philosophically significant concepts becomes the sole objective of philosophy. The name that pre-eminently comes in this connexion is Gottlob Frege since the analytic tradition in philosophy has been greatly influenced and guided by the Fregean assumption that language functions as an expression of thought. This is possible because language and thought form an indissoluble link. Frege thus finds that the crucial importance of language lies in expressing and communicating thoughts. From this idea of language expressing thoughts Frege further goes on to claim that meaning does not have distinct identity other than the expression of thought. In actual term, the sense or the meaning of a sentence as a whole is thus a thought or what Frege calls a proposition. The thought is a semantic content of a sentence that can be understood independently of how the sentence is used. However, the Fregean assumption that language expresses thought should not be viewed as a blanket expression. Frege in this respect takes special effort to show that there are definite logical mechanisms or devices operating in language through which thought reveals itself. Thought thus expresses itself through concepts, propositions, logical forms, functions, etc. Note, these logical devices are not external to thought but they are the basic structures of thought since through them thought is shaped and regulated for its proper expression. Consider the sentence, “Freud is a psychologist” which in the Fregean term, can be analysed into two parts—the functional part and the argument part. The linguistic expression “is a psychologist” is a function which Frege calls concept, and the name “Freud” comes under the argument part which designates an individual object. The expression “is a psychologist” is thus an incomplete part of the sentence which needs to be supplemented by a name designating an individual object for obtaining a complete sentence. Such a sentence is either true or false depending on the name suggested under the argument place. Instead of Freud thus there can be any other name that one can put, such as, Piaget, Parfit, or Newton.

Coming back to the idea that language expresses thought, it implies that there is a complex logical process involved which Frege tries to bring out by showing the way how thought interacts with language. In the context of the present inquiry, Frege's notion of concepts is especially important since as the bearer of thought they become the sole custodian of meaning. From our previous discussion, we know that concepts since they fall under predicate expressions are incomplete, such as, “is a psychologist” in our example. Now for the requirement of making a complete sentence, predicate needs to be connected to the object as cited in our example “Freud” which is by itself a complete expression. The linguistic correlate of the predicate expressing a concept Frege calls a concept-word. Since it is a linguistic expression by itself it is not a concept. It, on the other hand, stands for a concept. But the question is what is this concept that a linguistic expression designates? Further, what is its nature and where is it situated? None of these questions were unaddressed by Frege. First, it is amply clear that since concepts are designated by linguistic expressions, they cannot be the part of language. They are also not someone's idea. Nor can they be characterised as mental events. Frege rules out any such possibilities on the ground that concepts are objective. That means, they are abstract objects which exist on an independent ground. They are like Plato's forms constituting the world of their own which Frege (1967) calls “third realm.” Considering the objective nature of concepts, the questions that come to our mind are, what role do they play? And how are they related to the world? Concepts are indispensable to talk about objects. Objects which are distinguished by their respective properties fall under different concepts. To explain, our quoted example which says that Freud is a psychologist implies that Freud has the property of being a psychologist. This is same as to say that Freud falls under the concept “psychologist.”

Frege's notion of sense or meaning as the expression of objective thought thus laid down the foundation for a disembodied conception of meaning which had a far-reaching influence in the later development of analytic philosophy. Since philosophy is viewed as the engagement with the study of meaning or meaning of concepts, philosophers find the disembodied conception of meaning can be thus used as the most effective method to pursue their objective. This has led to the view that cognitive activities that we perform are mentalistic in nature. This, in other words, is to suggest that cognition is disembodied. Its disembodied nature comes as a result of disembodied notion of meaning which is presupposed at the background of all our cognitive activities. This is so because we see the world and understand it through language, that is, meaning. Hence, disembodied conception of meaning becomes central to our cognitive activities since it shapes our cognitive nature in a particular direction.

It is evident that the conceptual/thematic foundation of analytic philosophy was originally built on the Fregean assumption on meaning as the expression of thought. In his effort to do so, Frege never thought of the role that human body could play in shaping and structuring our thought. The same trend continued in the subsequent development of philosophy where the role of body was not recognised in relation to our cognitive activities, particularly, conceptualisation of meaning. The embodiment theorists point out this important lacuna which is crucial to the way philosophy is traditionally conceived in total isolation from its substantive physical basis. In this connexion, we can refer to some of the pioneering studies that have made the breakthrough in our understanding of the role of embodiment in philosophy. The study jointly carried out by Lakoff and Johnson (such as their work of 1998) and individually by Lakoff (such as Lakoff, 1987) and Johnson (1987, 1999) may be specially mentioned since the influence of these studies can be found evident throughout in the present work. In this connexion it may be also noted that Johnson's (1999) paper has been quite liberally used here because of its comprehensively posing the problem of embodiment in a nutshell.

In Johnson's (1999) view the disembodied conception of meaning is not an artificially construed notion. It is, on the other hand, originated in our common-sense view of the world and object. This view, since it is found to be plausible and is thus widely shared subsequently gets its justification in analytic philosophy expressed in terms of disembodied conception of meaning. In the common-sense view, the world consists of objects or things which are varied in nature and appear before us in different shapes, qualities, and so on. We thus encounter particular things like oranges, roses, horses, computers, etc. Each of these though is a particular thing, belongs to a class of things that share the same essential properties by virtue of which they are called by the same name. The common-sense view later finds its systematic exposition in abstract philosophical theories of meaning, such as, the one we find in Frege's notion of concept and object.

In the light of the common-sense view, the notion of meaning was traditionally construed in Richard Rorty's (1972) phrase, as a mirror or representation of reality in our mind. What is represented are those classes of things constituting the various categories of the world and the means through which they are represented as a system of science. These signs themselves are meaningless but they get meaning only through interpretation where interpretation is meant to be consisting of associating a sign with a category existing outside. Interpretation thus results into meaning. It is by establishing the right kind of relationship between a sign and the object that meaning is obtained. Such a conception of meaning cannot be anything other than being disembodied and literal. It is disembodied because the notion of meaning is formed independently of its bodily basis. It is also literal because meaning is strictly confined to the relationship between a sign and the object. It is not the object that gives the meaning, but it is the association established between the sign and the external world that becomes crucial for the concepts to be meaningful. Truth value of the sentence too will be accordingly determined by the correspondence between a sentence and the state of affairs existing in the world. Thus, for the sentence “The chair is green” to be true, it must satisfy the following conditions: first, the particular chair, as mentioned in the sentence, must belong to the class consisting of all chairs and, second, it must also belong to the class of green things. The semantic thesis that is derived from this relationship is that for a sentence to be meaningful it must be either true or false. This conception of meaning was widely shared particularly among logicians and philosophers of language and became the subject matter of semantics. Later in cognitive science this notion of meaning became the basis of mentalistic semantics where meaning was defined in terms of mental representation of external reality. Meaning since it is viewed as an outcome of arbitrarily establishing a relationship between a sign and the object existing in the external world, its nature becomes inevitably viewed as disembodied. The same disembodied view of meaning finds its reflection in philosophy of mind where the notion of mind is essentially conceived as disembodied in nature. This takes us to the next topic of discussion on mind.

### The Disembodied MInd

The idea of disembodied conception of mind goes back to the Fregean idea that language is the expression of thought. In this respect, the argument is given to show that thought has the same logical structure as that of language. Thought has the same subject–predicate structure along with other logical operators, such as, quantifiers and so on. Secondly, both language and thought share the same representational character which forms the strongest ground connecting the two. This structural similarity between thought and language makes Fodor (1975) to propose a hypothesis called language of thought. It may be defined essentially as a system of representation having analogous logical structure as that of language. Now the question is, what bearing does it have on mind? Mind has no separate existence apart from its various mental states. Mental states such as, “believing that P” or “desiring that P” are defined as computational states of the brain. The brain is thus thought of as a digital computer and the mind is accordingly viewed as the software of this computer. This constitutes the central idea of Fodor's computational theory of mind. It has two levels—the syntactic and the semantic. At the syntactic level, thought is viewed as purely mechanical computation whereas at the semantic level thought is projected as representational. The former leads to the latter which implies meaning correlates with form. But how is this possible? How do these two levels operate so that the harmony between the two is achieved? To put it more specifically: How does purely syntactic operation get its semantic interpretation? Block (1995) addresses these questions. According to him, syntax and semantics are intertwined with each other so that syntax mirrors semantics. In this intertwined mode a symbol gets its interpretation/meaning from its connexion with other symbols and the role that it plays in the network economy of the mind.

In this computational–representational conception of mind, Fodor argues that a system of symbols functions as mental representation because they are semantically interpreted symbols constituting the language of thought. Believing that Fido is faithful will imply having a sentence in the language of thought which is the mental analogue of the English language sentence “Fido is faithful.” The language of thought is a biologically fixed code. It can be compared in the way machine code functions as a part of the built-in structure of a computer. But the question arises, how is this biologically fixed code—the language of thought represented in the mind? Once again, this is understood in relation to the way computer operations are understood. There is a well-accepted standard procedure which shows how these operations are conceived and thereby represented in a computing machine. The specific mode in which they are represented is in terms of boxes or nodes in flow charts. There are three characteristics associated with this procedural mapping represented in terms of boxes. First, each box represents a particular function. Second, they are governed by the input/output structure. Third, these functions can be realised only when there is a right kind of causal structure involved. It is felt that in an analogous manner the mind too functions in the same way as a computing machine does. The functional organisation of the mind can be represented in a flow chart. Thus, different mental states like belief and desire form separate boxes and function in the same procedural way.

It is evident from the above discussion that the disembodied conception of meaning and the disembodied conception of mind are conceptually interconnected. This strong conceptual linkage between the two, as Johnson (1999) observes, has made considerable influence on what he calls “the first-generation cognitive science.” The idea of language as the expression of thought and mind as a formal device to compute that thought for various cognitive purposes became the principal source around which cognitive science was developed in its initial days. Cognition was thus seen as a purely mental phenomenon. As a result, it was a common tendency observed whether in cognitive science or in philosophy that the fact of embodiment was completely overlooked while explaining cognitive activities. True, activities like conceptualisation or reasoning are abstract, non-physical intellectual activities but it will be wrong if we look at these activities in isolation from their bodily basis characterised by sensory motor experiences. In fact, the two are so closely connected that the former, as Lakoff and Johnson argue, can be said to be the metaphoric extension of the latter. With this we now go to the second section where our main concern will be to give a philosophical justification for the bodily basis of our epistemic/cognitive persuasions. That is, the body has a fundamental epistemological function to play.

### II. Cognition and the Bodily Point of View: A Philosophical Justification From Merleau-Ponty

At the background of our earlier discussion on the disembodied perspective on cognition, in the second part our aim will be to introduce and to highlight the notion of embodied cognition. We will argue that cognition is not exclusively mental because it cannot be separated from its bodily basis. In other words, cognition as the claim made, assumes a bodily point of view which results into a new understanding called embodied cognition. The issues that need immediate attention are: what is body? How it is to be conceptualised? And what role does it play for making cognition embodied rather than disembodied in nature? From the nature of these questions, it is evident that the idea of embodied cognition requires, before probing into its empirical nature, philosophical/conceptual justification first. In this respect, Merleau-Ponty's attempt is a pioneering one since he has provided a full-length justification for the philosophical basis of embodiment in general.

We thus come to the discussion on the notion of embodiment. In this context the first and foremost issue that needs to be addressed is the traditionally conceived notion of mind–body dualism that constitutes the dividing line between embodiment and disembodiment. Since the former is understood in sharp contrast to the latter, the question that arises is: how does embodiment thesis resolve/accommodate the problem of mind–body dualism within its conceptual framework? This is a question which is fundamental to our understanding of embodiment as a thesis that looks at body as an expression of unity between mind and body.

### The Idea of Lived Body

In Merleau-Ponty's account, the unity between mind and body is not a superimposed metaphysical construct nor is it the result of certain invented mechanism connecting the two. On the other hand, for him, it is a felt unity—the unity that is experienced. In Merleau-Ponty's expression, this is called lived body where neither the mind nor the body is experienced as separated from the other. This inseparability between the two forms the crux of the state of embodiment in which consciousness is conceived as embodied implying that it is incarnated in a situation experienced by the body. Consciousness thus does not have any separate existence apart from body. This leads Merleau-Ponty to reject the disembodied conception of mind advocating a notion of pure consciousness whose only role to play is to watch the world as a spectator without getting involved in it. But Merleau-Ponty argues that this way of conceiving consciousness goes against our experiential evidence since I experience my body as subject which means that “I” is not different from “my body.” In other words, I am my body. Consciousness or subjectivity is embedded with the objective body and that makes a living dialogue possible between the subject and the world. The new notion called the body-subject thus replaces the widely prevalent idealist notion of subject of consciousness.

However, the question that arises in view of the idea of body-subject, how is body to be conceived and what constitutes the nature of body? In this context a strange attitude is found toward the nature of body. In both the idealist and the materialist tradition of philosophy body is viewed as an objective, meaning thereby, that body is like any other physical object in the world which is characterised by having a distinctive anatomical structure associated with a complex physiological mechanism built into the system for functional purposes of the body. Body as objective thus becomes a part of the subject matter of scientific study because all mental facts about a person are physical facts. But the anomaly found here is that the body cannot be conceived merely as an object—a physiological organism. This is the flaw committed by materialism which completely denies the importance of the mental and the subjective by reducing it to the physical and the objective. Idealism, on the other hand, takes a reverse turn by reducing the physical and the objective to the mental and the subjective on the ground that physical facts about a person can all be explained by subsuming it under mental facts. The same mistake is thus committed by idealism, the same anomaly is carried on by taking a reductionistic move. Note that despite their differences both idealism and materialism hold the same notion—the notion of objective body. The only difference is that in one case its primacy is celebrated and in another it is downgraded for being inferior to mind/consciousness. Both these trends have failed to see that such a separation between mind and body is not possible. As pointed out earlier, their unity is felt in our lived experience. In this respect, we can reconstruct form Merleau-Ponty the following arguments to establish the thesis how mind is embodied and how it, thereby, obliterates the distinction between subjective and objective, inner and outer, and mental and physical.

### First: Background Argument

Before we embark upon Merleau-Ponty's actual arguments concerning the way mind and body are embedded with each other that results into the state of embodiment, it is necessary to introduce first his idea of perception as a bodily phenomenon. The theory of body for Merleau-Ponty, cannot be given a separate place from the theory of perception since the former is a part of the latter. It is due to this inseparability that Merleau-Ponty calls perception as embodied. This becomes evident in my perceptual experience since I find that I perceive with my body. To say this is to mean that the position and movement of my body is crucial to the object that I perceive because to perceive an object will necessarily involve creating a perceptual field within which the object is perceived. Perception thus becomes perspectival, that is, depending on the physical orientation of the body I see profiles of the object. But as I shift my position from one side to the other side, I see the other aspects of the perceived object. The moot point that emerges here is that I can see only what my perspective allows me to see. This rules out the possibility of seeing the object from all sides in a given moment.

It will be, however, wrong to think from the perspectival nature of perception that it puts limitation to perception. Merleau-Ponty, on the other hand, would argue that contrary to what is thought about perspective as limitation to perception, it provides the real condition that characterises the way we encounter reality in our visual experience. Perspectival nature arises as a result of embodied nature of perception. The former is the expression of the meaning of the latter. The idea of perspective may lead to further misunderstanding. This is in connexion with the subject/object distinction traditionally held in the context of perception. This is because perspective may be viewed as the subjective component and the way in which the perceptual field as determined by it in the real world constitutes the objective component of perception. This is how the traditional idea of subject/object distinction in perception is reinforced with the idea of perspective. In Merleau-Ponty's response, this argument is artificially construed and lacks any basis solely on the ground that they are more apparent than real distinction. The reason is that both are expressions of the perceiving subject's state of embodiment in the world. Due to this in an act of a given perception both are found to be fused together. This allows Merleau-Ponty (1962, p. 132) thus to talk about the subject–object dialogue which means how both the subjective and the objective components contribute to make the sense perception possible for a subject. To approach it from the subjective side, perception is greatly influenced by the subject since it is processed and structured by the considerations which are intrinsic to the perceiver. Some of the important considerations that may be mentioned in this context are: first, perception depends on the spatial orientation and direction of the perceiver's body, second, an act of perception is always interest relative, that is, relative to what the perceiver is looking for, and, third, perception is accompanied by the perceiver's ability to focus on a particular visual field as against the initial anonymity and to look at it critically for the purpose of correct interpretation. But note that the subject alone does not matter in perception. An equally important role is played by the object too. This has been shown in *Gestalt* psychology which makes it evident that the importance of perceptual objects in influencing our perception is undeniable. Perception has its own inherent logic which is revealed in our perceptual experience. We thus see, for example, the visual world as consisting of meaningful structures. In a perceptual act a visual field is created which is directing me to focus on each of its parts and ascribes significance to them in the light of an overall pattern. Perception of colour is a good example of this where perception depends on the structure of my visual field which gets revealed due to the effect of the various shades of lighting.

Merleau-Ponty thus overcomes the barrier posed by the subject/object distinction. As a result, this allows the possibility of the dialogue between the subject and object possible. The reason for this being possible was that Merleau-Ponty changed the received conception of perception. Both empiricism and rationalism take one sided view of perception. In the empiricist standpoint perception is chiefly viewed as a passive process involved only in recording of sense data for the purpose of associating those sense impressions to form individual objects. Whereas, in the rationalist standpoint the active role played by the subject in interpreting those meaningless sense impressions becomes key to the explanation of perception. In the former case, perception turns out to be an automatic process which functions in the same way as a camera does. But in the latter case apart from recording sense data the essential task involved in making perception possible is to make interpretation of those data. In this respect, Merleau-Ponty's embodied theory of perception brings a radical change by introducing a new perspective on perception where neither the subject nor the object gets its primacy over the other. The idea that I perceive with my body summarises this new framework of thinking where perception becomes the bodily phenomenon. This amounts to say that trying to understand perception in abstraction is an impossibility on the ground that it cannot be uprooted from its material/bodily considerations. This is so because an important fact about perception is that a perceptual field is largely formed by the sensory motor structures and capacities of the body. This shows that the structure of perception does not have separate place from the structure of the body. The two cannot be differentiated which is expressed in Merleau-Ponty's observation when he says my body “is my point of view upon world” (Merleau-Ponty, 1962, p. 70). Considering the centrality of the position that embodied perception assumes Merleau-Ponty (1962, p. xi) thus finds perception to be the background for all acts. It is necessary to understand this for knowing the nature and constitution of the bodily subject and the way it functions. This, in other words, will be explicating the way embodiment becomes a reality in our lived experience. We will now present some of the key arguments of Merleau-Ponty to this effect showing how he arrives at the idea that embodiment is a fact.

#### First: I Am My Body

“I am my body” is an argument asserting that consciousness/self cannot be separated from body. This means that consciousness cannot be conceived without being embodied and thus as a result I “and my body” they become indistinguishable. But it may not be readily acceptable to everyone, particularly in view of the dominant belief that body is an object. Hence to say that I and my body are indistinguishable needs to be proved. True, body is an object. But this is not the only way to look at body. This is especially true in relation to the way I experience my body. In my lived experience I never comprehend my body as an object among other objects of the world. But why is this so? What is the compulsion behind it? This can be explained in the following way. Suppose I am sitting in my study and I want to shift my study chair near to the window for fresh air. I thus pull the chair and shift it from one place to another. But the crucial question that is raised here, can I move my body the same way as I can move the chair? Obviously, I cannot. The reason is that my body is always moved with me solely on the ground that I am my body. “I” and “my body” are not two different entities. That is why moving my body necessarily involves moving myself. “I am my body” is thus an expression of the fact that we are embodied beings.

In this context there is an adjoining argument revealing how my body assumes a unique position when I observe or perceive an object. To perceive an object involves that it is with the help of my body that I perceive the object and thus putting myself in relation to this object. This forms a perspective within which I perceive and locate the object. But most significantly, though it apparently looks trivial, I cannot perceive my own body in the way I can perceive the object. I cannot do this for a simple reason that I cannot have any perspective of my own body. This is so because my body provides the perspective that determines what is accessible to my vision. The lesson that can be drawn from here is that I cannot consider my body as one among several objects of the world. The body acts as a subject, in other words, it becomes an embodied subject. This is what is essentially felt in my lived experience. The unity that is felt here is already there and thus Merleau-Ponty describes it as pre-reflective or pre-thematic.

#### Second: Body Image

In the embodied approach body is never viewed as an inert substance that functions strictly according to a set of physical and chemical laws. This conception of body has been radically changed and being replaced by an alternative one where body is always viewed as the lived body characterised by its own intentions. In this interpretation the body is said to be equipped with a kind of latent knowledge due to which I develop a kind of self-awareness of my body. This has led me to understand that body is not a system of fragmentary parts but is an expression of composite unity. I discover that in my attitude toward body I find body as a totality stands for an expressive unity. Merleau-Ponty while speaking about this unity observes that my “whole body for me is not an assemblage of organs juxtaposed in space, I am in undivided possession of it.” I get to know form my experiences that all the parts of my body are “inter-related” in a unique way. As a result, they are “enveloped in each other.” This is the latent knowledge, as said earlier, that my body is constituted whose manifestation is found in the way the body functions as a harmonious system. It is, in other words, the awareness that my body is having of its own system. Now to suggest, that this is the result of a non-corporeal mind working from inside, as Descartes puts it, lacks any empirical or conceptual basis. Merleau-Ponty instead suggests that the way a body presents itself in our experience reveals an image which he calls “body image.” It is through this body image that I come to know “where each limb is” and how do they function in an interrelated manner within the bodily configuration. This body image that I have is not the product of conscious reflection but the latent knowledge which is built into my system (body) that accounts for the existence of body image. The idea of body image takes us to the next argument showing how body's behaviour is goal directed and, thereby, becomes intentional. Crucial to this is the role of the sensory motor mechanism of the body since that makes behaviour intentional.

#### Third: Bodily Intentionality

The discussion on how the lived body's behaviour is intentional requires the discussion to be carried out in the light of the two following questions, first, how does body acquire intentionality? And second, how does this intentionality express? The answer to the first question lies in the body itself. From the earlier argument we know that body is an expression of composite unity where all the parts of the body are never seen in isolation but as holding together giving rise to a unity. Further to this composite unity, I experience my lived body as an intentional unity or what Merleau-Ponty calls the body to be a “synergic system”—a system that itself turns out to be an expressive unity as distinct from a physical object. The body as a seat of intentionality is something that is pre-reflectively given. This can be best seen in the context where I am engaged in performing an action. I cannot perform an action successfully unless the relevant parts of my body cooperate in executing it. All these parts which are involved in the action are directed toward executing it. This is how they express intentionality. This partly answers the second question raised above. It can be further elaborated with an example from Merleau-Ponty (1962, p. 149) describing a scenario where I am sitting at my table and trying to reach the telephone. This involves a series of bodily movements which can be claimed to be following almost a sequential ordering starting right from the movements of my hand to the movements of my leg muscles. There are two conceptually important points emerging here. First is my desire to achieve certain results and second is the bodily movements which are so spontaneously coordinated that they can bring about the desired result. This is the reason why my entire bodily movement is found to be directed to the object. This simple fact shows the existence of bodily intentionality which is presupposed in every action.

The term “bodily intentionality” perhaps needs a little more elaboration since it conveys a rather generic meaning. If we go to the actual details of how bodily intentionality works, we will get to know the specific meaning of it as understood by Merleau-Ponty. Referring to his earlier cited example concerning my attempt to reach the telephone has been possible because of the coordinated effort initiated by the various parts of my body and their adjoining muscles. But behind this lies the actual storey according to which it is the interplay of motor nerves that make my action possible since it is the nervous system that forms the primary storage for body's decision and communication. The coordinated manner in which the body executes its functions is the result of the two-way process in which sensory motor nerves function. The afferent and efferent signals through which the brain gets information from the environment and, in turn, the instructions that it delivers to the rest of the body makes bodily intentionality possible. Bodily intentionality thus in essence is, what Merleau-Ponty calls, motor intentionality. With this, we now go to Merleau-Ponty's discussion of Schneider case.

#### Schneider: A Case Study

Merleau-Ponty's discussion of this case study brings out the seminal importance of motor intentionality. The following is the brief history of the case. Schneider is a veteran soldier who is a war victim of the first world war. His brain was partially damaged and due to this there was a change in his behaviour which was pathological in nature. The medical and psychological details relating to his body and the behaviour that was observed was studied and written down by the German psychologists Gelb and Goldstein. Merleau-Ponty carefully went through these details and used it for his own study to show what was wrong with Schneider's behaviour and why was it so. We can mention here the two major disabilities of Schneider.

First, Schneider cannot carry out any abstract movements without his eyes being open. An abstract movement is a kind of action which is performed only in an artificially constructed situation. As against this are concrete movements which are performed spontaneously in a normal practical or actual situation.

Second, Schneider lacks proper knowledge about how muscles are involved or used in the context of voluntary movements. It is a situation that indicates that he does not have kinaesthetic knowledge. This is observed from his own behaviour since he lacks the ability to specify the location of his various limbs in relation to one another. The same failure is observed as against the objects with which the limbs that are in contact.

However, as Merleau-Ponty allows, that some of the disabilities that Schneider is facing can be overcome. In this context, he mentions some of the actual possibilities that help Schneider to overcome it. Schneider, for example, can make abstract movements provided he is allowed to open his eyes so that he can observe the limbs that he is using to perform them. Also, he can move his whole body to achieve this purpose. He can do all these by thinking that as if he is in a practical situation. Regarding kinaesthetic knowledge, Schneider can improve the situation by rehearsing the bodily movements ordinarily performed in a practical situation. This will help him to overcome his disability because he can now recognise the parts of his body and the specific objects which are encountered by the body. Apart from these, Schneider is perfectly capable of doing his habitual actions like taking his handkerchief from his pocket to blow his nose or lighting a lamp by taking match from a box, etc. All these works are performed by Schneider without difficulty. As it appears, Schneider as such does not suffer from any damage either in relation to movement or to thought. He can move his body according to the instructions given to him. Similarly, he can carry out abstract thought and make hypotheses and act accordingly. But then the crucial question that arises is, what is it that Schneider is lacking?

In Merleau-Ponty's explanation what Schneider is lacking is motor intentionality which according to him is a form of operative intentionality. It is the most primitive form of intentionality through which consciousness makes a human world around us. Operative intentionality is thus pre-reflective or non-thematic and anti-predicative. It is reflected in normal subject's behaviour where body, as we have said earlier, functions as a unity holding all its parts together. In the process the body itself becomes expressive implying that the unity that it expresses is not a mechanical but an intentional unity. This is the way how our body experiences it and that is the reason why all our actions or bodily movements are enacted in a way where all the component parts of the body get involved as a whole. This is possible because of the body's built-in intentionality. Coming back to Schneider, the main source of his difficulty is that he does not experience his body as an intentional unity. As a result, he finds his body to be consisting of a series of isolated parts. This has been acutely expressed in the way he performs his actions. He lacks the ability to design or structure his actions. For this purpose, he develops an ideal formula to do the action. The other alternative that he uses is to make rehearsal as a preparation to do the action. Since Schneider cannot visualise the end, he cannot direct his movements toward it. Further to this there is a related difficulty that Schneider is facing. Since Schneider does not experience his body as an expressive unity he thinks that there is no one who is having this unity. The world for him thus loses its character. He does not find anything in the world which is emotionally exciting him. He lives in a fragmentary world which appears completely alien to him. Schneider is a classic case which shows what could happen when there is breakdown of motor intentionality.

Finally, what lesson does Merleau-Ponty offer? Alternatively, what is it that we can derive from his discussion on embodiment? We cannot fail to notice particularly from his discussion on bodily intentionality that embodiment has two overlapping aspects. The one is conscious subjective mental state, such as, my desire to achieve certain results like trying to reach at the telephone and the bodily movement which are spontaneously coordinated in a way that they can bring about the result. My entire bodily movement is directed to the object. This is the bodily intentionality presupposed in every action. The only way to explain this, as Merleau-Ponty argues, that consciousness relates itself to bodily activities. This speaks for the unity between consciousness and body.

### III. The Three Modes of Embodied Conceptualisation

The earlier section which has laid down the basic tenets of embodiment in general constitutes the philosophical foundation for embodiment. It seeks to show that the need for accepting the idea of embodiment is a conceptual necessity since without involving it understanding of our cognitive engagement with the world will be one sided and biassed. The received view of cognition characterised purely in mentalistic term is a glaring example to this one-sided biassed account. Embodied cognition thus defines the very essence of cognition which needs to be established not only at the conceptual level but also at the empirical level. The previous section has tried to do that at a conceptual level whereas the present section will provide justification at an empirical level. In this context the contribution of the second-generation cognitive science deserves to be specially mentioned. It provides decisive evidence showing the embodied nature of cognition. The present discussion will largely depend on these empirical/experimental findings for constructing its arguments for the bodily basis of cognition. This establishes a conceptual link between the second and the third sections.

The three modes of conceptualisation that will be discussed here, as mentioned at the beginning of the paper, are first, explaining the presence of categories, particularly, basic-level categories in human life and thought, second, colour concepts/categories and the process through which they are formed and, third, reconstructing the notion of reason to show how it functions as metaphor. Considering these objectives, a clarification needs to be offered. It may be noted that among these three the third one, i.e., the notion of reason as construed here is a departure from its accepted definition. At this stage thus to avoid confusion it requires immediate clarification. Conventionally, reason is viewed as a method—a logical tool to conduct an enquiry in a rational manner. This traditionally accepted picture is not denied here. But more than a method reason is itself perceived here as a source of conceptualisation. This is because reason cannot be separated from conceptualisation. In their study, Lakoff and Johnson have brought out this aspect of reason while substantiating their claim regarding embodied nature of reason. For this, they have considered spatial bodily relation concepts in order to show how reason is grounded on these concepts. These are the concepts by which we talk as well as conceptualise about space (Lakoff and Johnson, 1998, p. 28–37). Concepts like “up-down,” “front-back,” “in-out,” “near-far” are some of the instances of these spatial relation concepts. We characterise, for example, an object in terms of these concepts, such as, near or far in relation to something. Similarly, we characterise our bodies in terms of having front and back. These concepts come into existence in our vocabulary through our spatial experience while interacting with our physical environment. They are the concepts “that we live by in the most fundamental way” (Lakoff and Johnson, 1980, p. 311). It is because of their centrality that spatial relation concepts form the core of our conceptual system. This is evident from the way how these concepts are imaginatively extended to understand and interpret other concepts including the abstract ones. The imaginative extension that is made here involves a new dimension because the process through which this new understanding is achieved is a metaphoric process. It may be appropriate to say that metaphor functions here as a method of reasoning.

As we can see, conceptualisation is the result of a complex process since it entirely depends on such factors like our bodily orientation, the environment in which we live and the symbolic system that we inherit for negotiating with the world. Note that the totality that is found here is itself grounded in our embodiment. The embodied conceptualisation while it is structured and processed goes through certain constraints which are of different kinds, such as, “constraints set by our sensory motor system, our cognitive processing capacities, our interests and aspects of our environments” (Johnson, 1999, p. 99). This is what embodied conceptualisation means that replaces the traditional mode of conceptualisation as the basis for acquiring knowledge and cognition.

### Basic-Level Categories

It is a human tendency that we classify things and bring them under various categories based on common properties that they share. This is expressed in language in terms of general concepts, such as, chairs, colours, horses, etc. Categorisation can be thus claimed to be the result of the way we think. This constitutes the ground showing how we conceptualise through categories in the context of our life or thought.

Categorisation is traditionally viewed as abstract and disembodied human activity. But the question arises how far this traditional view can be legitimately held? In the embodied framework this traditional understanding has been questioned particularly in the work of Rosch (1978) where categorisation is shown to be dependent on the bodily orientation and capacities of the person who is doing the categorisation.

Traditional understanding of categorisation is essentially wrong. Categories are general terms or class expressions under which individual things are classified based on their common properties, such as all horses are brought under the general term “horse.” Common properties are thus taken to be the defining feature of forming a category. This is a position which has been universally accepted as what Lakoff (1987) calls “definitional truth.” But, as he points out, the fault lies here since what is claimed as universally true lacks any empirical basis. The position to which it has arrived is thus based on a priori speculation. The recent study in cognitive psychology brings out the fact that there are categories which Rosch calls basic-level categories that cannot be explained by the traditional view because they have properties which can only be explained in relation to human embodiment.

To start with, we go back to our original example—the word “chair.” It assumes its meaning by virtue of establishing a correspondence between the word “chair” and the object chair existing in the world. Meaning since it is defined in terms of correspondence it may be called the objectivist conception of meaning. Its objective nature follows from the objectivist conception of the world because the world is viewed as independent of mind and body. In this account categories, such as, the word “chair” or “tree” are formed, as the claim made, by following an objective procedure where a set of common properties shared by members belonging to a particular class decides the formation of a category. An important rejoinder needs to be added here. As Johnson (1999) points out “chair” belongs to a hierarchy of concepts where some of them are above and some of them are below the concept of chair in the hierarchy. In this hierarchical arrangement, “chair” is subsumed under the higher order category called “furniture” whereas particular classes of chairs, such as, “armchair,” “deckchair” are all subsumed under the higher order category called “chair.” Again, within the general category of furniture there are several classes of furniture to be found such as, “chair,” “table,” “bed,” etc. Note that they are in opposition to each other. As we know these categories or concepts in the hierarchy are formed based on a set of properties. These sets of properties, respectively, define what a concept of “furniture” and “chair” is. Further, the concept of “chair” though it belongs to the concept of “furniture,” has certain additional properties by virtue of which a chair becomes different from other furniture, such as, tables or beds. On the same ground if we go further down in the hierarchy an armchair will be found to have all the properties of a chair but also some additional properties that distinguish it from other types of chairs, such as, study chair or deck chair. In the traditional concept of category, the hierarchy associated with concepts is decided on the basis of objective properties of concepts and on this consideration “chair,” “tree,” or “car” are thus placed at the middle of this hierarchy.

The traditional picture of category, as mentioned earlier, has been seriously questioned and was overthrown in some of the recent studies in cognitive psychology by Rosch, Brent Berlin and others. The alternative explanation that Rosch offers may be briefly presented in the light of the account given by Lakoff (1987), Lakoff and Johnson (1998), and Johnson (1999). The first insight that Rosch offers is that the traditional explanation of categories being formed based on their objective properties is not tenable. This is particularly true of, as she claims, in the context of middle-level or what she calls basic-level categories, such as, chair, tree, or car. These basic-level categories as her finding shows can only be explained by taking the embodied perspective into account. To this effect, the earlier hierarchy is thus now replaced by a new hierarchy were basic-level categories are distinguished from superordinate categories (such as furniture, plant, or vehicle) and subordinate categories (such as armchair, sandalwood tree, or luxury car) on a fundamental ground involving our body, mind, and brain all three together. In short, it is the embodied perspective and not objective properties that matter in forming these categories. This will be evident if we look at the nature of properties by virtue of which the basic level categories are distinguished from superordinate and subordinate categories in the embodied framework. They are of various types, such as, “mental images, gestalt perception, motor programme, and knowledge structure” (Johnson, 1999, p. 89). All these though widely different from each other collectively constitute the state of embodiment which is crucial to the determination of these basic level categories. How these properties are individually responsible for determining the distinct nature of basic level categories can be listed out from the description provided by Johnson (1999, p. 89).

#### First

Mental images, it has been empirically found that a single incident of a mental image of an object will enable us to have the mental image of the entire class to which the object belongs. It is evident that a particular image of an object, such as, a chair, for example, can represent the entire class/category consisting of all types of chairs. Further the same mental image can also enable me to have the mental image of opposing categories, such as, beds and tables. In contrast to this, the possibility of having mental image of furniture as a category is ruled out on the ground that we can only have mental image of a particular kind of furniture, such as, chair or table but not furniture as a whole or category.

#### Second

Gestalt perception, we can identify a particular member belonging to the primary level category in terms of its perceived similarity with respect to its shapes that it shares with other members of the category/class. A chair or a car, for example, is recognised by its overall shape. However, the same is not possible for furniture or vehicle because they lack any such shape that we can ascribe to them. The perceived similarity of shape indicates the whole that leads to the perception of part i.e., a particular car. The reason is parts are contained within the whole.

#### Third

Motor Programmes, in our discussion on Merleau-Ponty, we have seen that as “neural beings,” to put it in Lakoff and Johnson's phrase (Lakoff and Johnson, 1998, p. 30) our actions are produced and controlled by sensory motor system that functions by following certain sequences. The neuronal structure of the body shows that our body is equipped with motor programmes that enable us to interact with objects at the basic level, such as, chairs, tables, beds, etc. But in the same way our body is not equipped with a similar motor programme for interacting with furniture as a category.

#### Fourth

Knowledge acquisition, the basic-level categories show marked differences in terms of knowledge that we acquire about them in contrast to what we know about superordinate categories. This is expressed from the detailed knowledge that we have, say, about cars in comparison to the few items that we know about vehicles. The basic level thus constitutes a body of systematically organised knowledge regarding its objects.

It is in the light of the above discussion that we can say, that because of its pervasive and effective use, the basic-level categories become fundamental to our categorisation. It thus truly gets its primacy over the two other categories, namely, superordinate and subordinate categories. As Lakoff and Johnson (1998) report that its primacy can be also found on two important grounds. The first is language since the existence of the categories can be found in the semantic vocabulary of the early history of language. The second is children's cognitive persuasion to know the world. As the evidence shows, they learn and use these categories initially for the purpose of exploring and interacting with the world. Later, the same practise continues with the adult mode of conceptualisation where we find that the use of these categories will enable us to function in the best possible way in our everyday life. Finally, no one can fail to notice that these basic-level categories are essentially body-based or better to say embodied in nature. The embodied perspective thus offers a full-fledged rejection of the traditional view based on the hypothesis that conceptual/cognitive categories are internal representations of an external reality. The philosophical claim made here is hypothetical because it has never been empirically substantiated. This takes us to the discussion of the specific form of categorisation, namely, the formation of colour categories.

### How Do Humans Form Colour Concepts or Categories?

Forming of colour categories is indeed a rich field of inquiry that clearly shows that the conceptualisation involved in the formation of these categories is embodied in nature. In short, colour category is an outcome of embodied conceptualisation. This will be revealed if we track the process of conceptualisation through which we arrive at colour categories. Note, the conceptualisation process is not independent from the empirical process involving neurophysiology of colour vision. It is only through such empirical study that we can find out the nature of conceptualisation that leads to the construction of colour categories. The empirical and the conceptual are the two parallel processes where the latter can be understood and explicated only in relation to the former. In the context of colour vision, the empirical process thus acts as a background to the conceptual process which in no uncertain terms shows that the nature of the conceptualisation involved here is essentially embodied. Apart from this, it further shows that construction of colour categories has certain far-reaching consequences. It brings out how this study of colour can bring a radical change to our approach to cognition in general. In this connexion, it may be argued that colour exemplifies the paradigm of a cognitive domain which neither exists independently of us as a pre-given phenomenon like any other natural objects of the world nor can it be said to be internally represented in our mind. Colour in this sense offers an example of the constructive nature of cognition which follows from the fact that colour itself is constructive.

Keeping this constructive nature of colour in view, Francisco Varela, Evan Thompson, and Eleanor Rosch, in their seminal study on colour perception, thus proposed what they have called an enactive theory of colour. The presentation to be made here will have a similar concern where the focus is to bring out the constructive nature of colour at two levels. The first is at the empirical level where the thrust will be to explicate the neurophysiological process that makes colour to be constructive. The second will be epistemological where the focus will be to show and to work out how the constructive nature of colour supports the view concerning the constructive nature of cognition.

Colour, as we see them, are always viewed in relation to things or objects in the world, such as, we say that the chair is green or that this is a red rose. But neither of these two are scientifically legitimate descriptions. The reason is that there is no such thing as a chair that is intrinsically green or a rose that is intrinsically red existing in the world. But, of course, we do say, that the chair is green or the rose is red. But how is this possible? It is possible because this is the way how we experience and understand the world where the world is not viewed independently of our body, brain, and mind. The utterance “the chair is green” thus makes sense because it is the expression of my implicit embodied conceptualisation. On the contrary, to see colour as existing independently in the world will not allow us to say that the chair is green on a scientific ground. In this context it is relevant to see briefly what the neurophysiological account of the process is that makes colour vision possible.

Perceiving of colour is the result of a complex process. We see a colour due to the interaction of the following factors which are of different nature: combination of different wavelengths of lights, colour cones and neural assemblies. Prima facie, it is the wavelength of light that strikes the process first since it is due to different wavelengths of lights that we see different colours like red, green, blue etc. However, for the process to be successful there are other crucial factors without which perceiving of colours will be well-nigh impossible. In this context the internal physiological structure of the eyes constitutes the most important part of the process. This brings us to the second factor relating to the retina and colour cones. Our ability to see colours is possible because our eyes contain photo-chemical substances whose response vary in accordance with different wavelengths. These wavelengths are classified and separated in different receptors which are of three types of cones situated in the retina. These three types of cones are the ones on which human colour vision depends. In general terms, each of these cones is characterised by having a particular kind of light sensitive chemicals known as photo pigment. Further each of these photo pigments selectively absorb wavelengths of different ranges. It is due to this reason that colour cones are found to be selective in their responses. The empirical evidence shows that one is sensitive to longer wavelengths whereas the other two are sensitive to medium and shorter wavelengths. The evidence of how wavelengths determine colour is observed in the context of chromatic and achromatic colours. Chromatic colours such as blue and green have one wavelength and that is the dominating one. In contrast to this are the achromatic colours which do not exhibit any one dominant colour or hue. These colours are white, grey, and black and they are governed by all wavelengths.

We come to the third factor or third level concerning neural assemblies in the brain. These neurons are the custodians of such cognitive tools like memories, concepts, words, etc. To speak sequentially, we have first seen that colour exists due to the light waves and second our ability to see colour is possible due to the functioning of colour cones in the retina. But the problem faced at the third level is specific in nature, that is, how do we get or form colour categories like red, blue or yellow? The process that makes it possible is through the work of neural assemblies. The three colour cones in our retina as the claim made, are connected to neural assemblies through which, as Johnson (1999, p. 86) puts it the “three fundamental opponent colour pairs: red-green, yellow-blue, white-black” are produced. Out of these three pairs, the first two (i.e., red-green and yellow-blue) stand for chromatic colours, whereas the last one (i.e., black-white) is a pair of achromatic colours.

From this bare minimum description of the way colours are arranged and classified indicates a new domain of human competence which was earlier unknown to us. It is the computational ability that enters at this stage of our colour vision represented by neural assemblies. It is thus widely realised and agreed that the entire structure of colour concepts/categories exhibited in our language use is the result of certain computational process performed in the brain. As a result, to put it in Johnson's (1999) phrase “a highly structured set of categories” is formed which is expressed in our language use. Even the sub-categories which are computed through basic categories are expressed in the linguistic modifiers. The category “green,” for example, assumes a chain of sub-categories which are all expressed through various linguistic modifiers, such as, “dark green,” “deep green,” and “light green” or “yellowish green,” and “bluish green,” etc. The same process is followed in other contexts, such as we notice that from the “combination of fundamental chromatic colours” (Johnson, 1999) the other chromatic colours are computationally derived.

It is amply clear that all these colour categories and their various permutations leading to sub-categories are possible not entirely due to light waves and colour cones in the retina but also important is the contribution made by neural assemblies. All these colour categories are evolved through a complex process. They are not pre-given-existing independently in the world and are perceived passively by us. On the other hand, they come to exist through a creative process involving both human body and brain particularly the active role that various sensory motor modalities play in this connexion. This brief discussion on colour perception brings out, first, the constructive nature of colour and second, the associated conceptualisation leading to the construction of colour categories embodied in nature. In the light of these findings, this discussion can now be extended to the epistemological level. Here our concern will be to show that embodied conceptualisation supports a new view of cognition.

It has been said at the beginning of the discussion on colour that conceptualisation of colour categories finally leads to a new cognitive domain where cognition is viewed as a result of active construction—a construction that is made by us. This has been brought out, as pointed out in our earlier remarks, in the study authored by Varela, Thompson, and Rosch. Their claim concerning the constructive nature of cognition is primarily based on the argument that our ability to see colour is possible due to the interplay of sensory motor modalities. Without their active involvement we cannot acquire the ability to see. The mapping of sensory motor modalities will show that they form a closely interconnected system where they influence one another. Now in this network of relationship if there is any failure or maladjustment, the entire system will get affected or disturbed. This has been shown in the work of Varela et al. in the light of a neurological case study of a colour-blind painter. It is through this case study that they have arrived at the two fundamental tenets connecting colour and embodiment. The first is, colour is constructive, and this nature follows from the way the visual system is structured. The second, colour perception functions within a totality, that is, in relation to other visual and sensory systems, or to put it in their expression it “partakes of both other visual and sensory modalities” (Varela et al., 1991, p. 164). With these clarificatory remarks, we now briefly present the case study of the colour-blind painter in the three successive phases.

#### The Case Study

The scenario of a colour-blind artist: a painter due to a car accident completely lost his ability to see colours. It was also noticed that the loss of this ability was not restricted to colour only, but its impact was widely found in the context of other sensory motor experiences, such as the way he experienced taste and sound. Further, as a result of the accident the painter's vision was severely limited, for example, he could see the world only within the narrow range of varying degrees of black, white, and grey. His power of imagination was also severely affected. This was expressed in his failure to imagine colours or to recollect what the various colours looked like. In the same way the possibility of dreaming in colours for him was ruled out. It was due to all these reasons that the painter was prevented from seeing the world as coloured. This brought a radical change in his experiencing of the world. He found everything around him as he reported, had a pale and dismal look. The surrounding environment for him was entirely changed. Everything was found to be “wrong, unnatural, stained, and impure.” This made a drastic change in his behaviour. He stopped enjoying life the way he used to do earlier like enjoying good food or music. Earlier he had the ability to visually represent musical notes into colour sequences which was lost due to the accident. But then the question arises what happened afterwards? A new storey starts.

#### The Changed Scenario

Later on, a strange phenomenon was observed. There was a remarkable change found in the painter's behaviour and his way of looking at the world. Earlier, his inability to see colour was a source of great agony to him. But now he finds that there is nothing agonising about it. Nothing is lost. This is possible because over the period he has adjusted himself to the new situation. Accordingly, his different mental traits and behaviour in general start accepting the new situation. In other words, his whole perspective changes and, as a result, he thinks and behaves like a night person. As he himself confesses that now “I love the night-time.” This is because, he finds that “it”s a different world: there's lot of space…it's a whole new world'. He no longer misses colour. It is expressed, in his own admission when he says “… now I don't even know it exists—it is not even a phantom.” This has led him to realise that “gradually I am becoming a night person” (Varela et al., 1991, p. 164).

#### The Explanation

The case study cited here raises two questions. How does the impairment of a particular sensory motor system (i.e., the ability to see colour) affects the other sensory motor systems like taste and sound? Second how does this change turn into a new form of behaviour and a new way of looking at the world? In their study, Varela et al. addressed these questions and offered their explanation. The essence of it can be summarised in terms of their following findings. First, as the case study shows the perceived world is not simply the given world taken for granted by us. It is, on the other hand, “constituted through complex and delicate patterns of sensory motor activity.” This is how the coloured world comes into existence through “complex processes of structural coupling.” Second, the problem starts if there is a change in the process and, as a result, certain patterns of behaviour can no longer be possible as the case study of the painter shows. Third, but this state of existence does not continue for a long period of time. It is a natural tendency of the body that it is always inclined to learn how to adjust to new “conditions and situations.” It adopts a new form of behaviour suitable to the changed situation. This result into attaining a new way of looking at the world which alternatively means change in “one's sense of the world.” As Varela et al. thus observe, “a different world is thus enacted” (Varela et al., 1991, p. 164).

The expression “enacted” used here has a deep significance indicating a new perspective on cognition. It is the constructive nature of cognition as mentioned earlier, that becomes the defining feature of the way cognition functions. This is revealed from the study of colour, particularly, from the case study of the colour-blind painter that provides an implicitly stated argument that there is no objectively universal view of the world available. That means, we do not cognise the world in the same way. This happens because of the way sensory motor modalities function in one's body. The cited case study is an illustration of the point revealing first, how sensory motor modalities function in virtue of which we get varied experiences of the various aspects of the world be it of colour, sound, or taste. Second, these modalities are so closely interconnected that if one particular modality, such as, colour is affected then other modalities too are affected. This is reflected in our experiencing of the world. That is, the world is not experienced in the same way as it was before in any other modalities like taste or sound. Third, the importance of environment is also crucial to our understanding of the world. We understand the world in terms of meaning. In the changed scenario, as mentioned in the second point above our understanding of the world also undergoes through a change. That means, I now understand the world in terms of a different set of meanings replacing the earlier one. Depending on the way we experience and understand the world we fix our meaning. But this meaning is not based on any objective relationship. It is, on the other hand, essentially to be viewed as embodied in nature. The colour-blind painter's view of the world changes because he finds himself to be in a different environment. Subsequently his body is adjusted to this new environment, and he starts loving it. He transforms himself to be a “night person.” This is a cognitive shift because he cognises the world in a different way and as a result a new set of meaning is invoked. This speaks for the constructive nature of cognition and colour provides the paradigm example of this nature of cognition. This is the reason why the conceptualisation of colour categories with which we have started our discussion ultimately ends in the discussion of constructive nature of cognition. The two are connected, where the latter is the epistemological off shoot that follows from the former. It is an epistemology of a different variety where cognition is viewed as an outcome of a process involving both body, brain, and the world. Cognition, so processed, is thus experienced as constructive in nature. It is now important at this stage to come to the final topic of our discussion concerning the nature of human reason and the way it is structured and functions within an embodied perspective.

#### Reason as Metaphor

At the beginning of this section while clarifying issues relating to reason it has been said that reason is a form of conceptualisation grounded on spatial or bodily related concepts. These concepts are central to our conceptual system because these are the concepts which are extended to understand and interpret the concepts of other domains of our life and experience. The principle by means of which this is carried out is through metaphor. It is a metaphor of a particular type which Lakoff and Johnson (1980) call conceptual metaphor. We will try to argue that metaphor assumes a different role here indicating that it is a method of reasoning which cannot be viewed as separated from its embodied nature. It thus indirectly questions the deep-seated belief that historically identifies reason with that of a formal structure as exhibited in algorithm.

It may be relevant to mention here that the notion of mind or reason as not being autonomous, that is, not independent of bodily capacities, has been widely shared in recent approaches to mind and cognition. In this respect, we can broadly identify the three such approaches which give rise to biologically phenomenologically and culturally inspired models for mind or cognition. Among these different models the notion of ‘extended mind’ introduced by Aund Clark (1997) and Clark and Chalmers (1998) may be specially mentioned since it has been an influential theme of discussion in the recent debate on mind and cognition. The idea of extended mind holds that the mind is not an insulated system working inside the skull. It is, on the other hand, a rich and an interacting system where external features outside the skin are equally crucial to the role to play in forming and shaping cognitive processes.

The present discussion on reason as metaphor has been carried out in three successive phases. First, the embodied nature of human reason and how it is developed and is blended with our other faculties for its functioning. Second, the role that image schemas play in connecting the bodily domain of reasoning with the abstract domain of reasoning. Third, the conceptual metaphor and the way it works in language.

#### Embodied Reason

To start with, it may be appropriate here to find out how the embodied view of reason is developed by humans at the early age of their life. In some of the recent studies on children's cognitive development it has been brought out that children at their early stage develop the idea of reason while carrying out their bodily activities. These are not mere bodily activities, they are, on the other hand, fit to be called embodied activities as found in such activities like perception, manipulation of objects, keeping body in a particular spatially oriented position, etc. All these embodied activities which involve sensory motor activities are performed by a new-born baby as early as when she is only 2 weeks old. As Daniel Stern's (1985) empirical/experimental studies on children show that it is through sensory motor activities that children at their infant age develop an understanding of the immediate situation around them. This is possible because they develop a sense of self—a sense of ownership. That is, I own such and such experiences. This experience of owning gives a child a sense of self identity. Further, the self that is witnessed is not a fixed permanent self. It is an emergent self that develops gradually in a child's life over a period starting from 2 weeks of her birth till 2 years and beyond. A significant aspect to be noted in this connexion is that it reveals certain startling facts about the child's/infant's cognitive development. First, the infant's experiencing of the world as the meaningfully structured one is pre-linguistic. It thus dispels the myth that language is the means through which the child comes to know that there is a meaningful world outside.

This, however, does not suggest that there is a notion of meaning without language. But if this is so what will be the nature of such a conception of meaning? This is not the place to go into the detailed discussion on the non- linguistic nature of meaning. However, a passing remark in the form of a clarification may be appropriate here. Experiencing the world as meaningful without the mediation of language means that the infant understands the world outside as an intelligible one. This is a non-conceptual understanding based on the infant's embodied experience. This makes a marked distinction between conceptual and non-conceptual understanding of the world to which we will come for discussion a little later. It will help in clarifying the non-linguistic nature of meaning.

Second, is the notion of self. It is the notion of an abiding self as the owner of all these different experiences establishes an identity between self and experiences. This forms a kind of self-identity in the child which is expressed in her behaviour as the experimental study shows. But this abiding nature of self should not be viewed as substantial or metaphysical self. Nor is it the result of a socialisation process. It is, on the other hand, an emergent self. It emerges, as Stern's study shows, based on our bodily experiences achieved through the operation of certain major sensory motor modalities. Self is an organisation built in an infant's life almost from the beginning through these modalities. As an illustration it may be mentioned here how babies discover schematic patterns—the commonly found structures, in different modalities, such as, vision, touch, and hearing. Similarly, there is a vast domain of emotional life or affective experience found in infant's life. As experimental studies show, the infant's experiencing of objects and people are invariably accompanied by their emotional responses, particularly, feelings like being happy, angry, or sad. For an infant, her world is thus characterised in affective terms. In continuation to this, there is an important fact regarding the emotive/affective domain of the infant's world. Stern argues that it is not that the infant's affective world consists of only certain discrete emotions like sadness or joy. Because and infant's experience may not be necessarily restricted to the standard range of emotions. It is not categorisation of a particular emotion, but the felt or phenomenal quality associated with the infant's experiencing of that emotion is important here. It is the felt experience of, say, sadness or joy that causes a particular state of arousal in the infant's mind which in turn motivates her to act in a certain manner.

The picture that emerges is that the affective or the emotive domain assumes a driving force in the infant's world. However, this does not imply that the emotive world is devoid of any cognitive import. The cognitive side of emotion can be evidently seen how, based on emotion, an infant gets involved in reasoning in order to arrive at the intelligible view of the world. Thinking and reasoning cannot be viewed in isolation, or it cannot be cut off from its experiential background because it involves various sensory modalities. In the context of rational activities, the nature of these modalities is different. As Stern (1985) says, they are not the usual modalities like sights, sounds, or touches since rational activities assume abstract representation of what the infant experiences at the primary level of perceptual experience. The sensory modalities that are involved here are “shapes, intensities, and temporal pattern.” They may be said to be “the more global qualities of experience” (Stern, 1985, p. 51–52) which constitutes the precondition of the infant's mental life. The central argument made here is that the infant's experiencing and recognising of objects and situations as significant in the world outside is the outcome of a complex process involving “cross-modal patterns of perceptual, emotional, and bodily interactions” (Johnson, 1999, p. 91). Alternatively, to put it in general terms, the infant's approach to the world provides the context that shows how reason, emotion, and sensory modalities are blended.

The same nature of blending continued if we look at the way the adult normal thinking is processed. In continuation, the observation made by Johnson is highly relevant. While referring to the phrase “the sentiment of rationality” used by William James, Johnson seeks to unpack the meaning of the phrase by describing what constitutes normal thinking. In his opinion, this very phrase summarises the essential structure of human thinking, that is, it is the result of “intimate intertwining of affective, sensory motor, and inferential structures” (Johnson, 1999, p. 93) presented in all aspects of adult thinking.

As a concluding remark to the discussion on cognitive development of infants, it can be claimed that an infant acquires her capacity to reason based on her embodied experience which she attains due to the interplay of certain bodily structures or modalities along with certain emotive elements mentioned earlier. Reason thus emerges out of embodied experience which in turn speaks for its embodied nature. An important fact to be noted in this connexion is that the infant's rational ability to negotiate with the world at this stage is carried out without the mediation of language since she does not have any access to use any linguistic skills at all. As a result, the understanding of the world that the infant acquires is non-propositional. This embodied non-propositional understanding is not lost even after the child acquires language. On the contrary, this mode of understanding forms the basic framework for the rise of higher order cognitive operations expressed through complex propositional and linguistic forms. There is an unbroken link found between embodied understanding and the propositional understanding. Now how is this link established? This is where the role of metaphors come which work through image schemas.

#### Image Schemas

At the beginning of the section while making introductory remarks it has been said that human reason does not have autonomy of its own. Instead, it has its basis in spatial relations concepts which are characterised in terms of bodily characteristics. The idea of image schemas is introduced here. They are schematic mental images expressing common patterns of various types of bodily orientations and interactions. However, these schemas owe their existence to sensory motor activities since without the latter the former cannot be formed. Embodied reasoning thus has its source in these image schemas without which reason will lose its bodily moorings. It will be evident if we see how crucial is the role that these schemas play to establish that abstract concepts and the reasoning accompanied by it cannot be separated from their bodily basis. The linkage between the two is established through these image schemas where the schemas themselves are metaphorically extended to the domain of higher order cognitive activities, such as, conceptualisation, understanding, reasoning, etc. The central point is that it is through these image schemas that embodied cognitive activities are structured. An illustration of an image schema will be helpful to understand how it works.

As an illustration we cite here the Source-Path-Goal image schema stated by Johnson (1999, p. 94). How it works may be presented sequentially in terms of the following description.

The everyday scenario: all our everyday voluntary activities are goal directed in the sense that they are performed in order to achieve certain purpose. Most of these activities are performed repeatedly by us and they follow a certain pattern, that is, they are originated form a certain point or source and they follow a path to realise the goal.

Examples: we walk to the refrigerator so many times in a day to get food from it. Similarly, to go out we go to the door to come out of the apartment.

In the same way we try to understand the movement of other people and objects appearing before our visual field. The same pattern is found here. There is an initial point from which they start and the destination to be reached in order to achieve a definite purpose.

It is also observed that the source-path-goal image schema like any other image schema is cross-modal. The same structure can be found with respect to all other modes of experience, namely, visual, tactile, and auditory. To follow the movement of a melody by using this schema is an example of how we understand auditory experience.

As we can see, source-path-goal schema is an imaginative structure that we develop based on our bodily experiences. These experiences are possible because this is the way the body is equipped with certain organs and mechanisms. They enable us to experience the world according to certain pattern. This would not be possible if we were not equipped with such bodily capacities like, for example, the way our bodies can freely move in space to carry out certain functions or the visual organs that enable us to detect a moving object along a certain path. All this is possible because this is how we are embodied. This makes it evident that the way we experience the world and the reasoning that we do for supporting it are enacted through the various image schemas which are available due to the way we are embodied.

The same thesis has been confirmed in recent research in cognitive science particularly in cognitive psychology and cognitive semantics (Lakoff, 1987; Turner, 1991; Gibbs, 1994). The findings of this research show that human cognition is a complex product where mental phenomena are analysed and processed in terms of bodily phenomena. That means, cognition is not just a mental phenomenon alone. It has a bodily basis which is revealed in our experiencing and conceptualising of all aspects of mental phenomena, such as, memory, knowledge, reason, and logic. Their bodily basis can be shown to be evident, if we see how we understand and characterise these mental activities in terms of certain image schemas. The notion of reasoning, for example, exhibits a process which is analogous to the source-path-goal image schema discussed earlier. Reasoning also like goal-directed bodily movement follows a certain path starting from an initial point with a purpose to reach the destination. If we look at the structure of reasoning, it starts from one idea which can be called the initial location. Then it moves to the next idea—the second location and finally it reaches at the conclusion which is parallel to what is called destination in the context of bodily movement.

One may think that this is an artificial way of looking at reason which prevents us to see what the real nature of reason is. But such an observation will be incorrect. The reason is conceptualisation of all higher order mental/cognitive activities are implicitly based on the way we understand our bodily activities with the help of image schematic concepts. Through their use the image schemas make embodiment perspective prominently present while pursuing our cognitive activities. From our earlier discussion we know that the embodied perspective offers an integrated framework where the emotive/affective domain has given an important place. In this context, image schemas are especially important. It is due to the presence of this affective domain that image schemas assume a new meaning enabling us to understand how important these schemas are in relation to a new way of understanding the world and of ourselves. These image schemas are embedded with emotive significance which motivates us to act in a certain way.

### Conceptual Metaphor

We come to the final phase of our discussion where our task is to show how metaphor works in connecting the two domains of conceptualisations. This consists of showing how bodily based conceptualisations are used as the basis for understanding the conceptualisation made in an abstract domain—a pattern of understanding in the light of which we analogously understand what the latter means and how it functions. These two though are different domains they are connected through a metaphor or a conceptual metaphor where the former is called the source domain and the latter is the target domain. The very term the “source domain” suggests that it provides the conceptual resources in the patterns of which the target domain needs to be interpreted.

Considering this, the role that metaphor or a conceptual metaphor plays may be viewed as, what Johnson (1999, p. 94) says “a conceptual mapping of entities and structure from a domain of one kind (the source domain) to domain of a different kind (the target domain).” A conceptual metaphor is thus a connecting link between the two domains. It offers a new way of looking at the target domain. This is possible because we metaphorically understand the workings of the domain by extending the conceptualisation made of the source domain. However, this is not to replicate the source domain on to the target domain. It is, on the other hand, achieving a new understanding of the reality. This, indeed, is the work that a metaphor does in its capacity as a creative device. The two important things to be noticed from this discussion are, first, metaphors work as a system of reasoning enabling us to see the imaginative dimension of reason and second, conceptual metaphors cannot be isolated from their bodily basis characterised in terms of image—schematic concepts. This indicates the embodied nature of conceptual metaphors. In continuation to this discussion, we now come to the discussion of concrete cases of conceptual metaphor where our purpose will be to see how they function in connecting the source and the target domains of conceptualisation. This will result in creating a new meaning and a new understanding of the abstract concepts of the target domain.

#### The Three Illustrations

We will now present here the three illustrations of conceptual metaphor. The three cases are all about abstract concepts relating to our moral/ethical nature. Through this presentation, it will be shown that the nature of these moral concepts are defined by means of metaphors. In this context our discussion will mostly follow Johnson (1993) and Lakoff's (1996) account. However, the citations made here are from Johnson's (1999) work.

#### Moral Strength

It is a commonly used ethical concept in the light of which we describe one's ethical character. A person having moral strength means he/she has a strong will to overcome the desires and inclinations leading to do the wrong things. Johnson argues that the way we understand the notion of moral strength and formulate it in terms of moral will is based on our understanding of physical strength. This understanding and the subsequent conceptualisation of physical strength is mapped on to the abstract domain relating to the idea of moral strength. The former can be thus called the source domain, the latter is the target domain where the two are connected through the relation of metaphor. That is, we understand the meaning of the expression “moral strength” metaphorically based on the meaning of physical strength. My body may have the capacity to do certain things, such as, lifting of heavy weight or the capacity to run for 10 miles at a stretch.

Alternatively, if I lack this, I will not be able to do this. This is how the two notions physical strength and physical weakness are understood in terms of our body's ability to do certain things. The notion of moral strength which belongs to a different domain is thus understood in similar terms. But the only difference is that instead of strength and weakness understood in bodily terms they are now understood in terms of will. Moral strength or weakness are accordingly judged whether I have the sufficiently strong will to overcome my unethical nature internal to my own self. There is always a conflict within me—the conflict between the higher self and the lower self. The higher self is a moral self, whereas the lower self is the immoral self, guided by lower instincts, passions, wicked desires, and temptations. A person having the moral strength is the one who has the firm will to overcome the dark forces of the lower self.

#### Evil as Darkness and Light as Spirituality

Johnson has discussed these two metaphors which are widely used by us since they most appropriately bring the sharp contrast between moral and immoral activities. It is a prevalent practise observed in almost all human culture that we see darkness as evil and light as truth. This is reflected in our use of language, such as, “ADG has so many dark sides in his character” or “The only hope is that ADG is trying to overcome it.” The darkness here means the evil and immoral streaks of ADG's character. Similarly, the expression “light” signifies the removal of darkness and revealing of truth which is spiritual and moral in nature. The source of this metaphor lies in our felt experience of darkness. We fear darkness because we commonly associate darkness with something that is evil, sinful, and immoral. Note that we form this association, as Johnson comments not because we cannot properly see things in darkness but the very feeling that we are engulfed by it that matters when we see darkness as evil. Our conceptualisation of evil is thus grounded in a significant way on this feeling—the feeling of being in darkness.

#### Moral Nurturance

Analogous to physical nurturance, Johnson introduces the concept of moral nurturance. In a way parent–child relationship a child is physically nurtured by her parents so that she can grow and develop in a desired manner. Now this idea of physical nurturance of a child by her parents can be extended, as Johnson argues, to her moral nurturance. This is again an extension from the physical domain to the moral domain where the conceptualisation of the former is mapped on to the latter domain. The meaning of moral nurturance must be thus understood as a metaphor. The way a child for her physical nurturance gets care, love, and affection from her parents to grow; in a similar way, for her moral nurturance she needs care, love, and empathy for her moral development or moral growth. It is only at the background of moral nurturance that what Aristotle calls the second nature of a person can be formed.

At the end, we can say that metaphor does not replace reason, but it certainly functions as a reason. It is through the implicit use of metaphor that we can understand the abstract domain of our thought, that is, how abstract concepts acquire their meanings based on bodily based concepts. The discussion on metaphor as reason thus brings out the fundamental nature of reason under the two aspects. First, the imaginative role that reason plays and second, the autonomy of reason can be seriously questioned on the ground that reason is essentially embodied in nature.

This brings us to the concluding remarks: with the idea of human embodiment, philosophy takes a new turn—a conceptual shift from its disembodied to the embodied perspective. Consequently, the distinction between science and philosophy can no longer be rigidly maintained since both will be complementary to each other without losing their distinctive identity.

This is where the present paper goes beyond the perspective laid down by such authors like Johnson, Lakoff, Varela et al., Rosh and others. For these authors, as it appears cognitive science offers answers/solutions to philosophical questions/problems. In this sense, cognitive science has a foundational importance for philosophy because it can bring a phenomenal change in philosophers' approach to cognition and how it is to be viewed. With this new change philosophers' idea of epistemic engagement as a central philosophical concern undergoes through a drastic revision. It is now largely viewed in terms of empirical engagement, that is, providing the empirical basis to knowledge and cognition.

The present paper, on the other hand, takes a different strand. Accordingly, it argues that the epistemic engagement has a distinctive philosophical nature which cannot be replaced by cognitive science. But then what role does cognitive science play in philosophy? On this issue the present paper takes the position that the role that cognitive science plays in the context of philosophy is to corroborate the conceptual/epistemic claims that philosophy makes. In specific terms, corroboration here is meant to be empirically confirming the philosophical claims. This way philosophy can retain its distinctive identity without surrendering it to cognitive science. This can be evidently seen in the c0ntext of the present inquiry where a clear demarcation is drawn between the conceptual and the empirical. In the light of this distinction Merleau-Ponty's phenomenological model in this paper is thus viewed as providing the conceptual foundation for embodiment whereas cognitive science provides the empirical confirmation to the conceptual claims made in the earlier philosophical part of the paper. In this sense, the conceptual and the empirical in this inquiry form a totality offering a new way of understanding cognition and cognitive mechanisms underlying it.

## Data Availability Statement

The original contributions presented in the study are included in the article/supplementary material, further inquiries can be directed to the corresponding author/s.

## Author Contributions

The author confirms being the sole contributor of this work and has approved it for publication.

## Conflict of Interest

The author declares that the research was conducted in the absence of any commercial or financial relationships that could be construed as a potential conflict of interest.

## Publisher's Note

All claims expressed in this article are solely those of the authors and do not necessarily represent those of their affiliated organizations, or those of the publisher, the editors and the reviewers. Any product that may be evaluated in this article, or claim that may be made by its manufacturer, is not guaranteed or endorsed by the publisher.
